# Perceived partner substance use, genetic predispositions, and their associations with problematic alcohol use, emotional well-being, and relationship quality

**DOI:** 10.1017/S0033291726103237

**Published:** 2026-03-24

**Authors:** Megan E. Cooke, Sally I-Chun Kuo, Erin Lumpe, Fazil Aliev, Sarah J. Brislin, Kathleen K. Bucholz, Grace Chan, Danielle M. Dick, Howard J. Edenberg, Chella Kamarajan, John Kramer, Weipeng Kuang, Samuel Kuperman, Vivia V. McCutcheon, Zoe Neale, Martin H. Plawecki, Bernice Porjesz, Jill A. Rabinowitz, COGA Investigators, Jessica E. Salvatore

**Affiliations:** 1 Department of Psychiatry, Robert Wood Johnson Medical School, Rutgers University, Piscataway, NJ, USA; 2 Department of Psychology, Rutgers University, Piscataway, NJ, USA; 3 Department of Psychiatry, Washington University School of Medicine, St Louis, MO, USA; 4 Department of Psychiatry, School of Medicine, University of Connecticut, Farmington, CT, USA; 5 Department of Psychiatry, Carver College of Medicine, University of Iowa, Iowa City, IA, USA; 6 Robert Wood Johnson Medical School, Rutgers Addiction Research Center, Rutgers University, Piscataway, NJ, USA; 7 Department of Medical and Molecular Genetics, Indiana University School of Medicine, Indianapolis, IN, USA; 8 Department of Biochemistry, Molecular Biology, and Pharmacology, Indiana University School of Medicine, Indianapolis, IN, USA; 9 Department of Psychiatry & Behavioral Sciences, SUNY Downstate Health Sciences University, Brooklyn, NY, USA; 10 Department of Psychiatry, Indiana University School of Medicine, Indianapolis, IN, USA

**Keywords:** alcohol use, cannabis use, GxE, rGE, romantic partners

## Abstract

**Background.:**

Romantic relationships are important contexts for substance use and emotional well-being. We tested the hypotheses that (i) genetic predispositions for alcohol consumption would be positively associated with partner substance use, (ii) partner substance use would moderate genetic influences on one’s own alcohol outcomes, and (iii) partner discordance in substance use would be associated with lower emotional well-being and relationship quality.

**Methods.:**

Analyses included 2,357 participants (M_age_ = 51.4, 58.2% female) from the Collaborative Studies on the Genetics of Alcoholism. Focal measures included participants’ reports of their own and their current partner’s past-year substance use (frequencies of alcohol use, heavy drinking, drunkenness, cannabis use, and nicotine use), emotional well-being, and relationship quality. Participants’ genetic predispositions were indexed with genome-wide polygenic scores for alcohol consumption (PGS_Alc_). Participant–partner substance use discordance was calculated as the difference between the participant’s and their partner’s use for each substance use measure, separately.

**Results.:**

Participant PGS_Alc_ was not significantly associated with partners’ perceived substance use. Frequent perceived partner alcohol use and heavy drinking significantly amplified the association between PGS_Alc_ and alcohol use or drunkenness. Frequent perceived partner drunkenness and cannabis use significantly attenuated the association between PGS_Alc_ and heavy drinking or frequency of alcohol use. Participant–partner discordance for several substance use measures was significantly associated with lower emotional well-being and relationship quality, controlling for participant and partner substance use main effects.

**Conclusions.:**

The results highlight the importance of partner substance use in etiological models of alcohol use, emotional health outcomes, and relationship quality.

## Introduction

Marriage and marriage-like relationships are important contexts for understanding the development and persistence of hazardous patterns of alcohol use and alcohol use disorder (AUD) (Leonard & Rothbard, 1999; McCrady & Flanagan, 2021). Two-thirds of American adults are married or cohabit with a partner (Pew Research Center, 2019), and among married couples, nearly 80% of leisure time is spent with one’s spouse (Genadek, Flood, & Roman, 2016). A consistent body of research has demonstrated that spouses/partners in marriage-like cohabiting relationships resemble one another in their patterns of alcohol use (Leonard & Rothbard, 1999; Mushquash et al., 2013; Windle & Windle, 2014), and that immediately following the development of AUD in one partner, the risk for AUD in their spouse is substantially increased (Grant et al., 2007; Kendler, Lonn, Salvatore, Sundquist, & Sundquist, 2018).

It is also well recognized that patterns of alcohol consumption and vulnerability to problematic alcohol use reflect a dynamic interplay between psychosocial and biological influences across the lifespan (Boness, Watts, Moeller, & Sher, 2021; Sher, Grekin, & Williams, 2005; Young-Wolff, Enoch, & Prescott, 2011). Genetic factors associated with alcohol use may contribute to individuals’ selection into or creation of marital environments (i.e. gene-by-environment correlation), such as selecting a partner who uses more substances or in a hazardous manner (Salvatore, Prom-Wormley, Prescott, & Kendler, 2015; Scarr & McCartney, 1983). Likewise, in light of evidence that permissive environments can serve as contextual triggers for the expression of underlying genetic predispositions (i.e. gene-by-environment interaction or GxE) (Shanahan & Hofer, 2005), we hypothesize that a partner’s substance use may moderate the associations between genetic predispositions and alcohol use behaviors. More specifically, we expect that having a partner who uses substances more frequently or in more hazardous patterns (e.g. heavy drinking) may be a contextual trigger that strengthens the associations between an individual’s genetic predispositions and their alcohol use behaviors.

Above and beyond the level of one’s own and their partner’s substance use, several studies have also identified partner discordance in patterns of substance use as an important contextual factor in understanding the nature of the associations between substance use and couple functioning (Derrick, Wittkower, & Pierce, 2019; Homish & Leonard, 2007; Leonard, Smith, & Homish, 2014; Meiklejohn, Connor, & Kypri, 2012; Wiersma & Fischer, 2014). The results from this prior work largely emphasize that *discordance* in partners’ patterns of substance use is associated with relationship outcomes, with greater discrepancies between partners associated with poorer relationship outcomes in domains of satisfaction and stability. In contrast, less is known about how partners’ discordance in substance use may be associated with individual-level indicators of health and well-being (Birditt et al., 2024). What is known suggests that dyadic configurations of substance use are important predictors of individual health outcomes, although the direction of effects is mixed. In one study, Birditt and colleagues found that spouses who are both current drinkers outlive spouses who are concordant non-drinkers (Birditt et al., 2024). Yet, concordant drinking at high levels is not uniformly associated with more favorable individual outcomes. For example, husbands’ blood pressure was higher when husbands and wives drank more, even when controlling for the husband’s drinking (Birditt, Turkelson, Polenick, Cranford, & Blow, 2022). This mixed pattern of effects highlights the need for further research and the importance of considering dyadic effects to understand individual well-being.

Our goal in the present study was to investigate the nature of the associations between partner substance use, genetic predispositions, and alcohol use behaviors, and how discordance in partners’ substance use is related to individual emotional well-being and relationship quality. We tested the following hypotheses:

1. Those with higher genetic predispositions for alcohol consumption (as indexed with genome-wide polygenic scores, which capture genetic risk across the genome) would also have partners with greater substance use.
2. Partner substance use would moderate genetic risk for alcohol use on phenotypic alcohol use behaviors (frequency of use, drunkenness, and heavy drinking).
3. Participant–partner discordance in substance use (alcohol, drunkenness, heavy drinking, nicotine use, cannabis use, and drug use) would be associated with lower emotional well-being and relationship quality.

In exploratory analyses, we examined whether these associations differed as a function of the focal individual’s age/generation, sex, parenthood, and race/ethnicity.

## Methods

### Sample

Data came from the Collaborative Study on the Genetics of Alcoholism (COGA), which is described in detail elsewhere (Dick et al., 2023). The analytic sample for the present study included participants from previous assessment waves who were recontacted and interviewed beginning in 2019 as part of the COGA Lifespan Study. The Lifespan Study consisted of two groups of participants: (1) individuals in early midlife between the ages of 30–40 years (born between 1982 and 1993; *N* = 1,114), and (2) individuals in later life aged 50 or older (born before 1974; *N* = 2,186). All participants in the Lifespan Study completed the Semi-Structured Assessment for the Genetics of Alcoholism (SSAGA) (Bucholz et al., 1994; Dick et al., 2023) for DSM-V clinical interview and self-report questionnaires, and had previously been genotyped (Johnson et al., 2023; Lai et al., 2019). Embedded within the SSAGA interview, participants reported demographic characteristics of their current partner and their perceptions of their partner’s substance use. Analyses were limited to participants who reported being married, living as married, engaged, or dating exclusively and therefore reported on their spouse/partner.

### Measures

#### Demographic information

Participant sex was recorded as male or female. Parenthood status was based on participants’ reports of whether they had any biological children.

#### Alcohol use behaviors

Participants reported how frequently in the past year they (1) drank any alcoholic beverages (i.e. alcohol use frequency), (2) got drunk (i.e. drunkenness), or (3) had five or more drinks in a 24-hour period (i.e. heavy drinking). Response options for these questions were ‘everyday’, ‘5–6 days a week (nearly everyday)’, ‘4 days a week (200–259 days)’, ‘3 days per week (150–199 days)’, ‘2 days per week (100–149 days)’, ‘1 day per week (50–99 days)’, ‘3 days per month (36–49 days)’, ‘2 days per month (24–35 days)’, ‘1 day per month (12–23 days)’, ‘6–11 days per year’, ‘3–5 days per year’, ‘1 to 2 days per year’, or ‘never’. Response options for each question were recoded to create separate semicontinuous measures of alcohol use frequency, drunkenness, and heavy drinking as days in the past year by calculating the average number of days covered by each categorical variable.

#### Nicotine use

Participants reported whether they had used cigarettes, cigars, tobacco pipe, or tobacco hookah, chewing tobacco, or electronic cigarettes in the past year. Past year nicotine use was coded as a binary variable.

#### Cannabis use frequency

Participants who had ever used cannabis reported how many times they had used it in the past year. Participants who had never used cannabis were coded as 0 times.

#### Other illegal drug use

Participants reported whether they had used any of the following illegal drugs: cocaine, stimulants, sedatives, opiates, PCP, hallucinogens, solvents (like glue, paint, or white-out), combinations (like ice or speedballs), or others (like GHB or MDMA) in the past year. Past year illegal drug use was coded as a binary variable.

#### Emotional well-being

Current emotional well-being was assessed using the National Institutes of Health Toolbox Emotion Battery (NIHTB-EB) (Salsman et al., 2013). The battery assesses five major domains: psychological well-being, stress, self-efficacy, social relationships, and negative affect. Psychological well-being assessed an individual’s satisfaction with their life, the feeling that their life has meaning, and positive affect. Stress assessed how unpredictable, uncontrollable, and overloaded an individual perceives their life to be. Self-efficacy assessed an individual’s belief in their capacity to manage problems. Social relationships assessed emotional support, friendship, instrumental support, loneliness, perceived hostility, and perceived rejection. Negative affect assessed an individual’s feelings of anger, hostility, physical aggression, apathy, fear, anxiety, somatic arousal, and sadness/depression. We used T-scores for each of those domains, as described by Salsman et al. (2013), in our analyses.

#### Relationship quality

Participants rated how much their partner makes them feel loved and cared for, makes too many demands on them, is willing to listen when they need to talk, is critical of them, they can depend on their partner, there is tension between them, they can open up to their partner, and they have disagreements or conflicts (8 items total) on a 5-point scale from ‘not at all’ to ‘a great deal’ (Salvatore, Aggen, & Kendler, 2022; Salvatore et al., 2015; Spotts, Prescott, & Kendler, 2006; Zlotnick, Kohn, Keitner, & Della Grotta, 2000). Items were recoded such that higher scores reflected better relationship quality, and were summed to create a relationship quality score (Salvatore et al., 2022).

#### Partner demographics

Participants reported their partner’s sex (male or female), age, and the length of their relationship.

#### Perceptions of current partner substance use

Participants reported on how often in the past 6 months their partner drank alcohol in a typical week (i.e. alcohol use frequency), got drunk (i.e. drunkenness), drank four (for females) or five (for males), or more drinks in a 24-hour period (i.e. heavy drinking), used nicotine products, used cannabis, and used any of the following drugs; cocaine, stimulants, sedatives, opiates, PCP, hallucinogens, solvents, combinations, or others. Partner measures of substance use were recoded to parallel the self-report of the corresponding measure in the participant described above.

#### Participant–partner substance use discordance index

We created measures of participant–partner substance use discordance by subtracting the partner’s use from the participant’s use within each substance use measure. The absolute value of these difference scores was carried forward into analyses so that larger scores indicated a greater discrepancy between the participant’s substance use and their partner’s substance use.

#### Polygenic score

We used summary statistics from the largest GWAS of alcohol consumption (drinks per week) at the time of analysis (Saunders et al., 2022) to create polygenic scores for study participants representing an individual’s genetic predisposition to alcohol use (PGS_Alc_). For individuals most genetically similar to reference panels from Europe (EUR-like), we used PRS-CS (Ge, Chen, Ni, Feng, & Smoller, 2019) to calculate the PGS using weights from the results of the GWAS based on EUR-like samples. For individuals most genetically similar to reference panels from Africa (AFR-like), we used PRS-CSx (Ruan et al., 2022) which boosts power from the results of the smaller AFR-like GWAS by meta-analyzing these with the results of the larger GWAS of EUR-like samples. We took an integrative data analysis approach for analyses that used the PGS. Specifically, we regressed PGS on the first 10 ancestral principal components (PCs), separately within each genetic similarity group, and saved the residuals. The residualized PGS from these models were then pooled and carried forward in subsequent analyses.

### Analytic plan

Analyses were preregistered at osf.io/63zwm. We used Pearson and tetrachoric correlations to assess partner–participant concordance for the continuous and binary substance use measures, respectively. For Hypotheses 1 through 3, we ran mixed-effects models accounting for familial clustering using *lmer* (for continuous outcomes) and *glmer* (for binary outcomes) from the *lme4* package (Bates, Mächler, Bolker, & Walker, 2015) in R. We included participant sex, age group (early midlife versus later life), current marital status (married versus not), and length of relationship as covariates for all models. For models that included PGS_Alc_, a variable indicating genetic similarity group (EUR-like versus AFR-like) was also included as a covariate. For models that included discordance scores as predictors, a variable indicating whether the participant or partner had the higher score, as well as the main effects for the corresponding participant and partner substance use measures, were included as covariates. We performed FDR corrections within each research question using the Benjamini–Hochberg method (Benjamini & Hochberg, 1995) and the p.adjust function in R.

After testing the key hypotheses described above, we examined if the above results differed as a function of participants’ age group (midlife versus later life), sex (female versus male), and parenthood (yes versus no). To balance Type I error concerns while also systematically examining these potential effect modifiers, we took an omnibus approach to test these interactions by including all interaction terms in one model and using model fit indices to determine if there was an improvement in model fit with the interaction terms. As a sensitivity analysis, we also included the interactions between PGS and the genetic similarity group when testing moderation models. If including the interaction terms resulted in an improved model fit, we reported the findings from the significant interactions within the improved model.

## Results

### Descriptive statistics

Descriptives of the final analytic sample (N = 2,357), broken down by sex and age group, are shown in Table 1. A subset of the analytic sample provided DNA (N = 2,182) and completed the NIH Toolbox Emotion Battery (NIHTB-EB; N = 865). Participants who completed the NIHTB-EB were more likely to be older, belong to the later life age group, have been in their relationship for longer, and be parents compared to participants who did not complete the NIHTB-EB. Completion of the NIHTB-EB was not associated with sex, marital status, or any of the participant or perceived partner substance use variables.

### Participant and partner substance use concordance

Substance use concordance between participants and their perceptions of their partner’s substance use was as follows: (i) frequency of alcohol use: r = 0.36 (95% CI: 0.33, 0.40), (ii) frequency of drunkenness: r = 0.07 (95% CI: 0.03, 0.11), (iii) frequency of heavy drinking: r = 0.20 (95% CI: 0.16, 0.24), (iv) nicotine use: r = 0.49, (v) frequency of cannabis use: r = 0.31 (95% CI: 0.28, 0.35) and (vi) illegal drug use: r = 0.71. Participants and their partners were highly concordant (93.04%) for illegal drug use (see Supplementary Table S1), resulting in too little variation within couples to run the subsequent planned analyses.

**Figure 1.**
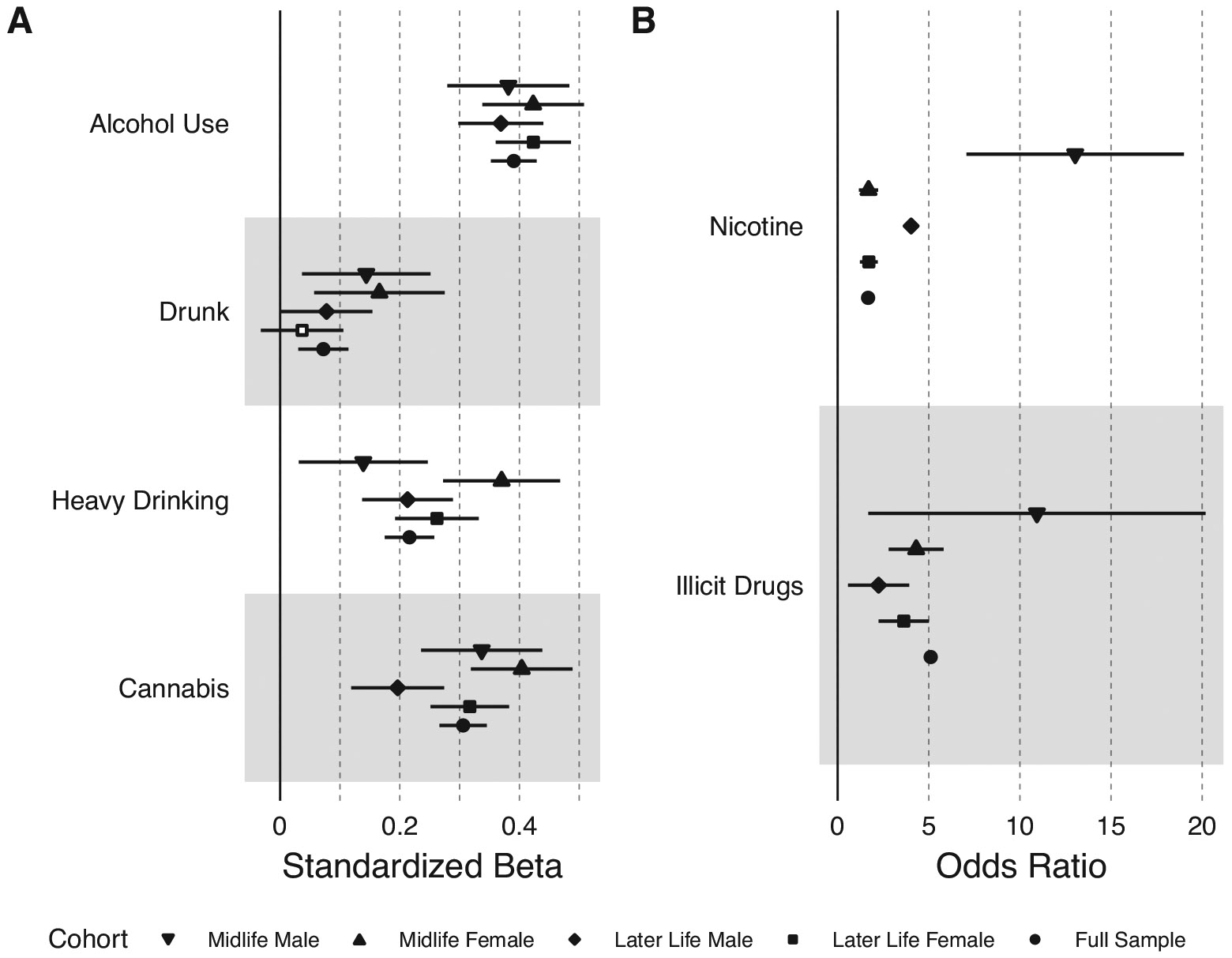
Concordance between participants and their partners on substance use measures. Estimates are shown for the full sample and stratified by sex and age group. Midlife is ages 30–40 and later life is ages 50 and older. Plot A shows the substances that were measured continuously. Plot B shows the substances that were measured as binary variables. Filled-in circles represent a statistically significant association at p < 0.05.

Figure 1 shows the results of the associations between participant and perceived partner substance use, stratified by sex and age group (see Supplementary Table S2 for full model results). Larger effect sizes indicate more similarity between the participant’s substance use and their perceived partner’s substance use. Participant sex modified some of these associations (see Supplementary Table S2). There was a significant interaction between sex and participant substance use in predicting perceived partner substance use for frequency of alcohol use (b = 0.20, 95% CI: 0.12, 0.27, FDR-corrected p = 8.08 × 10^−7^), frequency of heavy drinking (b = 0.32, 95% CI: 0.24, 0.41, FDR-corrected p = 9.80 × 10^−14^), and cannabis use (b = 0.31, 95% CI: 0.23, 0.39, FDR-corrected p = 9.80 × 10^^14^), such that female participants more closely resembled their perceived partner’s substance use than male participants. Participant age group also modified the association between participant and perceived partner cannabis use (b = 0.11, 95% CI: 0.03, 0.18, FDR-corrected p = 0.015), such that the association was stronger for individuals in midlife.

### Gene–environment correlation analyses

Participant PGS_Alc_ was not significantly associated (ps > 0.05) with their perceived partner’s alcohol, nicotine, or cannabis use. See Supplementary Table S3 for full model results.

Adding sex, age group, genetic similarity group, and parenthood status as moderators to the nicotine use model resulted in an improvement in model fit (X^2^(4) = 9.89, p = 0.04). There was a significant moderating effect of sex on the association between PGS_Alc_ and perceived partner’s current nicotine use (b = 0.319, 95% CI: 0.065, 0.572, p = 0.014) such that in female participants, higher PGS_Alc_ was associated with a greater likelihood of their partner having used nicotine in the past year, while in male participants, higher PGS_Alc_ was associated with a greater likelihood of their partner not having used nicotine in the past year. The inclusion of sex, age group, genetic similarity group, and parenthood status as moderators did not result in an improved model fit for any of the alcohol or cannabis models (*p*s > 0.05).

### Perceived partner substance use as a moderator of genetic predispositions

As a prelude to the interaction effect models described below, we ran models quantifying the main effect of participant PGS_Alc_ on each of the participant alcohol outcomes. Participant PGS_Alc_ was significantly associated with the participant’s frequency of alcohol use (b = 0.062, 95% CI: 0.020, 0.104, p = 0.004), drunkenness (b = 0.040, 95% CI: 0.0004, 0.080, p = 0.048), and heavy drinking (b = 0.055, 95% CI: 0.014, 0.095, p = 0.009).

**Figure 2.**
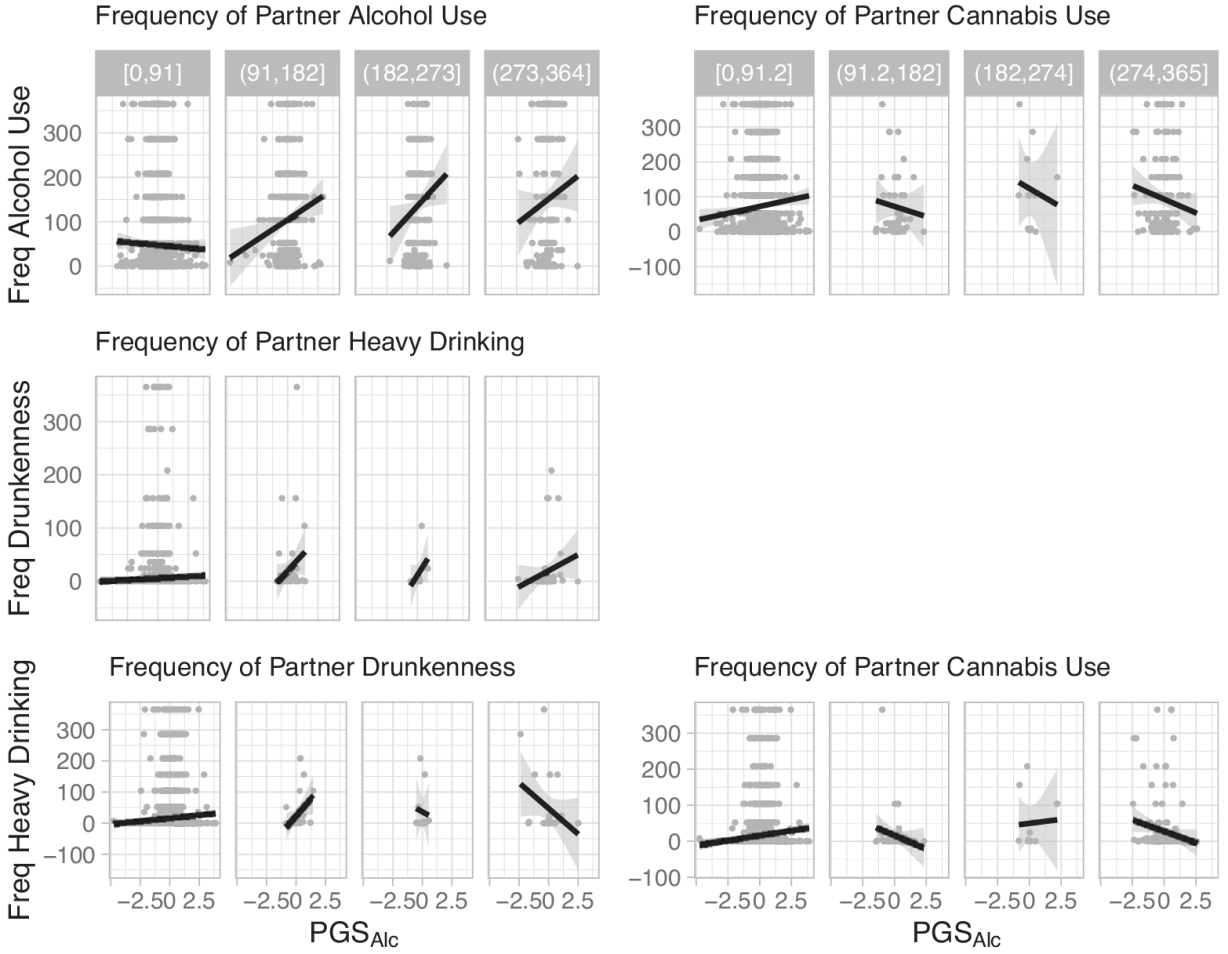
Conditional plots of moderation effects. Note: Conditional plots of the observed data for the statistically significant two-way interactions for participant alcohol use outcomes as a function of PGS_Alc_ and partner substance use. The data were split into four groups of equal range on the partner substance use variables. The range defining each of these groups is shown in gray at the top.

As depicted in the Figure 2 conditional plots, perceived partner substance use moderated participant genetic predispositions to predict several alcohol use outcomes. Perceived frequency of partner alcohol use moderated the association between PGS_Alc_ and participant frequency of alcohol use (b = 0.090, 95% CI: 0.048, 0.132, FDR-corrected p = 0.0004), and perceived frequency of partner heavy drinking moderated the association between PGS_Alc_ and participant frequency of drunkenness (b = 0.053, 95% CI: 0.011, 0.096, FDR-corrected p = 0.050). For these effects, the association between PGS_Alc_ and participant frequency of alcohol use or drunkenness was stronger when they reported that their partner drank alcohol or engaged in heavy drinking more frequently.

Perceived frequency of partner drunkenness moderated the association between participant PGS_Alc_ and participant frequency of heavy drinking (b = −0.045, 95% CI:−0.011, −0.079, FDR-corrected p = 0.049). In contrast to the other models, the association between PGS_Alc_ and participant heavy drinking was attenuated when they reported that their partner was drunk more frequently. Likewise, perceived frequency of partner cannabis use moderated the association between participant PGS_Alc_ and participant frequency of alcohol use (b = −0.044, 95% CI: −0.008, −0.080, FDR-corrected p = 0.050) and heavy drinking (b = −0.057, 95% CI: −0.022, −0.092, FDR-corrected p = 0.011). The associations between PGS_Alc_ and participant alcohol outcomes were attenuated when they reported that their partner used cannabis more frequently. See Supplementary Table S4 for full model results.

In exploratory analyses, adding sex, age group, genetic similarity group, and parenthood status as moderators to the interaction between PGS_Alc_ and perceived partner heavy drinking in the heavy drinking model resulted in an improvement in model fit (X^2^(4) = 20.265, p = 0.0004) with parenthood being the only significant moderator (b = 0.237, 95% CI: 0.095, 0.380, p = 0.001, Supplementary Figure S1). The inclusion of sex, age group, genetic similarity group, and parenthood status as moderators to the interaction between PGS_Alc_ and perceived partner nicotine use in the heavy drinking model resulted in an improved model fit (X^2^(4) = 18.576, p = 0.001) with sex being the only significant moderator (b = 0.174, 95% CI = 0.086, 0.262, p = 0.0001, Supplementary Figure S2).

### Participant–partner substance use discordance, emotional well-being, and relationship quality

**Figure 3.**
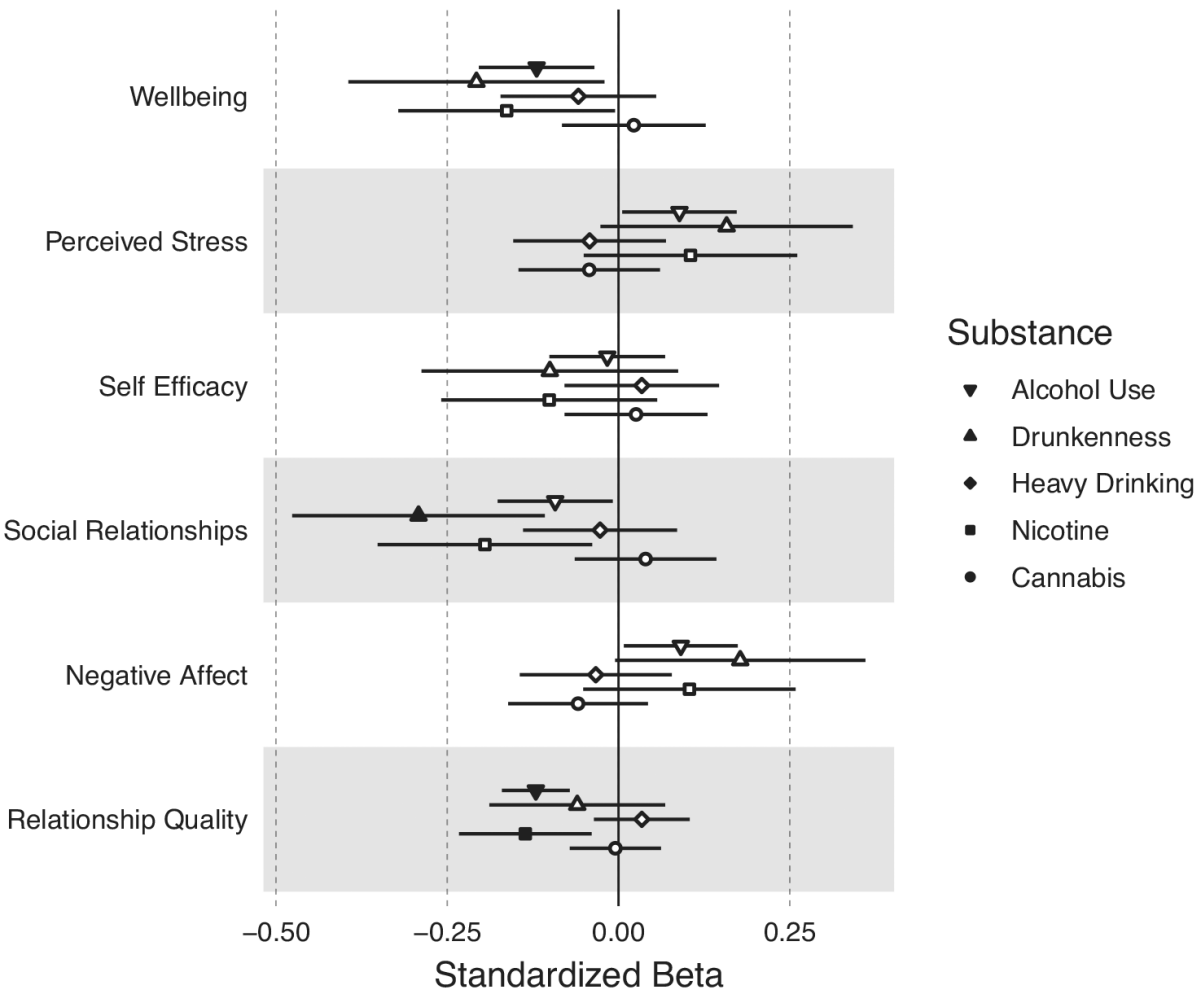
Associations between substance use discordance within couples and emotional well-being and relationship quality. Discordance between participant and partner on their substance use was measured as the absolute value of the difference between participant substance use and partner substance use. Larger discordance scores therefore indicate a greater discrepancy between the participant’s substance use and their partner’s substance use. Filled-in circles represent statistically significant associations at an FDR-corrected p-value <0.05.

Figure 3 shows the associations between participant–partner substance use discordance on facets of emotional well-being and relationship quality. Higher discordance in frequency of alcohol use was associated with lower well-being (b = −0.120, 95% CI:−0.204, −0.035, FDR-corrected p = 0.045) and relationship quality (b = −0.121, 95% CI:−0.170, −0.071, FDR-corrected p = 5.85 × 10^−5^). Higher discordance in frequency of drunkenness was associated with lower social relationships (b = −0.292, 95% CI:−0.476, −0.108, FDR-corrected p = 0.030). Discordance in past year nicotine use was associated with lower relationship quality (b = −0.136, 95% CI:−0.233, −0.039, FDR-corrected p = 0.045). Discordance in frequency of heavy drinking or cannabis use was not associated with any emotional well-being or relationship quality measures (uncorrected ps > 0.05). See Supplementary Table S5 for full model results.

Adding sex, age group, genetic similarity group, and parenthood status as moderators did not result in an improved model fit for any of the above models (ps > 0.05).

## Discussion

In this report, we sought to evaluate gene–environment correlation and interaction processes for alcohol use behaviors in the context of romantic relationships, and the associations between partner discordance in substance use with emotional well-being and relationship quality. In contrast to our hypothesis, we did not find evidence that polygenic predispositions for alcohol consumption were associated at a statistically significant level with perceived partner substance use behaviors. These null effects were unexpected in view of prior evidence from a Swedish national sample that individuals with a parental history of AUD, who we can infer have a higher genetic (and environmental) loading for the disorder, are more likely to marry AUD-affected spouses (Salvatore et al., 2019), as well as prior evidence that polygenic scores for alcohol consumption were associated with a spouse’s drinking in non-clinically ascertained US and European samples (Clarke et al., 2021; Otten & Mandemakers, 2023). Although we cannot fully explain why our findings diverge from these earlier reports, sample size may be a contributing factor. A post hoc power analysis indicated sufficient (80%) power to detect effects (standardized betas) of 0.062, but the largest effect we observed was b < 0.01. Itis also possible that within the clinically ascertained COGA sample, the associations between participants’ polygenic predispositions and their partners’ substance use were diminished by broader background familial factors that often co-occur with AUD (e.g. lower socioeconomic status or family disruption; Grant et al., 2015) and are also associated with assortment and partner selection (Erola, Härkönen, & Dronkers, 2012; Salvatore et al., 2019; Schuckit, Tipp, & Kelner, 1994). In addition, by focusing on questions about partners’ concurrent alcohol use behaviors, we may not have captured the full range of a partner’s lifetime patterns of alcohol and substance use behaviors. For example, we would expect polygenic associations to be attenuated if a participant with a higher polygenic score reported on concurrent alcohol use behaviors in a partner who was in abstinent remission from AUD.

We found mixed evidence for our hypothesis that perceived partner substance use moderates the associations between polygenic predispositions and alcohol outcomes. Perceived partner alcohol use and heavy drinking moderated the influence of participants’ polygenic risk scores on frequency of alcohol use and drunkenness, respectively. These findings were consistent with our hypothesis that perceived frequent alcohol use or heavy drinking by the partner would strengthen the association between a participant’s polygenic score and their alcohol use or drunkenness. In contrast, perceived partner drunkenness moderated participants’ polygenic risk scores to predict heavy drinking in the opposite direction than anticipated. We observed that individuals with higher genetic predispositions for alcohol consumption engaged in less frequent heavy drinking when they reported that their partner got drunk more frequently. Although this effect runs counter to our original hypothesis, we recently observed a similar pattern of partner GxE effects in a population-based Finnish twin sample (Stephenson et al., 2024). In that study, latent genetic influences on binge drinking were attenuated as partner alcohol use increased.

Interestingly, perceived partner cannabis use was the most pervasive moderator of genetic predispositions on all alcohol outcomes. The associations between the alcohol consumption polygenic score and participant alcohol use, drunkenness, and heavy drinking were weaker when a partner reportedly used cannabis more frequently. This may be the result of substance substitution (Subbaraman, 2016), whereby participants with partners who use cannabis frequently choose to use cannabis instead of alcohol despite a genetic predisposition to alcohol use. As a set, these results underscore the salience of partner characteristics in modifying genetic influences on a variety of drinking patterns, and are consistent with the broader literature that romantic relationships are important social contexts that shape individual health behaviors (Birditt et al., 2022; Holt-Lunstad, Robles, & Sbarra, 2017). Our findings add a genetic perspective to this understanding, demonstrating that genetic factors play only a limited role in alcohol use behaviors when one’s romantic partner is drunk or uses cannabis more frequently. These results, which suggest that individual diatheses such as genetic predispositions vary in salience depending on the couple context, highlight the importance of considering partner alcohol and cannabis use as part of preventive intervention efforts for alcohol use (Epstein & McCrady, 1998; McCrady & Flanagan, 2021).

Our exploratory analyses also revealed that sex and parenthood each modified a two-way gene-by-partner substance use interaction effect. Visualization of these three-way interactions suggested that perceived partner substance use as a moderator of polygenic predispositions tended to be less precisely known in one group compared to the other due to data sparseness. Owing to this, we caution against interpreting these results as evidence that the effects differed between groups (e.g. early midlife versus later life, parents versus non-parents, etc.).

We also found evidence consistent with our hypotheses that partners tended to resemble one another in their substance use, and that having a partner whose substance use is different from one’s own is associated with lower emotional well-being and lower relationship quality. Similar to reports from clinically ascertained and community-based samples (Agrawal et al., 2006; Ask, Rognmo, Torvik, Røysamb, & Tambs, 2012; Low, Cui, & Merikangas, 2007; Treur, Vink, Boomsma, & Middeldorp, 2015), COGA participants and their romantic partners were moderately correlated for alcohol use frequency and had a relatively strong concordance for nicotine use and illegal drug use. Interestingly, partners’ resemblance for frequency of drunkenness was relatively weak, indicating that while participants perceived that they and their partners drink at similar frequencies, they perceived less congruence in their drunkenness. We also found evidence that sex and age group moderated partner resemblance. Compared to male participants, female participants reported that they resembled their partners more in their frequency of alcohol use, heavy drinking, and cannabis use. In opposite-sex relationships, female partners are often considered the ‘barometer’ of the relationship (Floyd & Markman, 1983), though more recent work has challenged this idea (M. D. Johnson et al., 2022), and partners who have concordant substance use patterns tend to be more satisfied and stable (Homish, Leonard, & Cornelius, 2008; Kulak, Heavey, Marsack, & Leonard, 2025; Leonard et al., 2014). In view of this, we speculate that females may be especially motivated to see their partner’s substance use as similar to their own. Our finding that midlife individuals reported greater resemblance between their own and their partner’s cannabis use compared to later-life individuals may reflect the more permissive norms and attitudes that midlife individuals have towards cannabis (Campbell, Twenge, & Carter, 2017).

Consistent with previous studies (Crane, Testa, Schlauch, & Leonard, 2016; Cranford, Floyd, Schulenberg, & Zucker, 2011; Homish & Leonard, 2007), we also found evidence that partner discordance in alcohol and nicotine use was linked to lower relationship quality. Discordance was also associated with several indicators of participants’ individual functioning. Alcohol use and drunkenness discordance were associated with lower self-reported well-being and lower scores on the social relationship scales, which are inclusive of social support, companionship, and social distress. Thus, mismatched substance use patterns appear to be associated with both poorer relationship outcomes and more broadly with poorer emotional well-being. This maps onto a growing literature that demonstrates that couples’ drinking patterns are associated with key individual health outcomes, including blood pressure and mortality (Birditt et al., 2022, 2024), and demonstrates an association with an individual’s emotional health.

The results from this study should be considered in the context of its limitations. First, COGA is a sample enriched for AUD risk, and accordingly, the pattern of effects observed here may not be generalizable to low-risk populations. Second, our measures of partner substance use came from COGA participants. We recognize that such reports may be biased (Kendler et al., 1991). Encouragingly, others have reported moderate associations between partner perceptions of problem drinking and self-reported drinking problems (Rodriguez, DiBello, & Neighbors, 2013). Nonetheless, we recognize that both perceptions of a partner’s substance use and the partner’s actual substance use can have independent and interactive associations with relationship outcomes (Rodriguez et al., 2013). Third, this report is based on cross-sectional data, and for this reason, we cannot make causal claims about the observed associations. Fourth, the definitions for heavy drinking when participants reported on their female partner were four drinks in 24 hours (compared to five drinks for all other participants and partners), which may capture a less intense version of heavy drinking. Fifth, even in our sample enriched for risk, there was very little variation between partners in illegal drug use, which precluded testing hypotheses using these measures. Finally, we recognize that there are different ways to calculate and analyze PGS. Our approach of residualizing PCs within genetic similarity groups and pooling has been used in previous studies (Barr et al., 2022; Choi et al., 2025) and allowed for increased power to detect significant effects, particularly in moderation models.

In summary, the results from this study in a sample enriched for familial risk for AUD offer novel insights into the interplay between genetic predispositions and partner characteristics on alcohol use behaviors. An especially novel aspect of our findings was that perceived partner cannabis use and drunkenness attenuated the associations between genetic predispositions and heavy drinking. These gene-by-environment interaction effects were not confounded by gene–environment correlation, as individuals’ genetic predispositions were not associated with their reports of their partners’ alcohol, cannabis, and nicotine use at a statistically significant level. We also expanded upon earlier insights that partner discordance in substance use is associated with lower relationship quality and found that partner discordance in alcohol use and drunkenness were associated with lower individual well-being and indicators of broader social relationship functioning. Partner discordance in nicotine use was likewise associated with lower social relationship functioning. Taken together, these findings emphasize the importance of considering dyadic partner effects in the etiology of alcohol use behaviors and emotional health.

## Supplementary Material

Supplement 2

Supplement 1

**Supplementary material.** The supplementary material for this article can be found at http://doi.org/10.1017/S0033291726103237.

## Figures and Tables

**Table 1.** Descriptives of the sample

	Early midlife		Later life	
	Male	Female	Male	Female
	% or M (SD)	% or M (SD)	% or M (SD)	% or M (SD)
Total N	328	493	658	878
Age	32.9 (2.8)	32.9 (2.6)	62.4 (8.8)	60.5 (7.8)
Genetic similarity^a^	(n = 291)	(n = 453)	(n = 619)	(n = 819)
AFR-like	22.7%	24.1%	5.0%	16.5%
EUR-like	76.3%	74.2%	83.8%	83.2%
Current relationship status				
Married	57.3%	61.3%	81.3%	78.8%
Widowed	0.3%	0.0%	1.2%	1.9%
Separated	1.5%	3.7%	2.6%	4.0%
Divorced	2.4%	4.5%	7.0%	8.1%
Never married	27.7%	19.7%	1.8%	2.4%
Living as married	10.7%	11.0%	6.1%	4.8%
Relationship quality^b^	26.7 (4.7)	26.4 (5.8)	25.6 (5.1)	25.7 (5.6)
Parenthood	52.1%	67.1%	90.7%	93.3%
Participant past year substance use				
Ever alcohol use	97.6%	97.2%	96.8%	96.0%
Freq any alcohol^c^	95.7 (104.6)	57.7 (79.9)	91.2 (120.7)	68.6 (108.2)
Freq drunk^c^	16.5 (55.1)	7.1 (31.4)	7.6 (37.4)	6.0 (34.1)
Freq heavy drinking^c^	35.0 (76.7)	13.8 (50.0)	22.9 (68.3)	14.9 (56.5)
Nicotine use	36.3%	15.4%	23.9%	17.5%
Cannabis use^c^	102.4 (188.7)	45.6 (129.7)	39.6 (115.8)	19.9 (78.3)
Illegal drug use	3.7%	1.8%	2.4%	1.5%
Emotional well-being^d^	(n = 105)	(n = 154)	(n = 251)	(n = 355)
Psychological	49.1 (10.0)	49.9 (8.7)	49.4 (7.8)	49.9 (8.4)
Stress	51.7 (9.7)	53.1 (9.8)	48.4 (8.5)	49.4 (8.7)
Self-efficacy	51.9 (8.9)	48.2 (9.4)	49.5 (8.5)	50.3 (8.8)
Social relationships	46.2 (10.8)	48.3 (10.0)	48.1 (8.9)	48.9 (10.0)
Negative affect	51.2 (11.4)	53.1 (11.2)	48.7 (10.0)	49.5 (9.3)
Partner characteristics				
Gender of partner				
Female	95.4%	4.3%	96.0%	1.9%
Male	3.0%	94.3%	0.9%	94.9%
Partner age	32.8 (4.6)	35.5 (5.1)	60.4 (10.0)	62.9 (9.0)
Age difference	0.2 (3.7)	^2.6 (4.3)	2.1 (5.9)	^2.4 (5.1)
Length of relationship	7.5 (4.8)	8.5 (5.1)	28.6 (16.1)	28.6 (16.0)
Partner substance use				
Freq any alcohol^c^	70.1 (86.5)	96.7 (105.0)	70.1 (96.6)	103.0 (128.0)
Freq drunk^c^	6.1 (26.1)	9.7 (42.4)	4.7 (30.7)	12.3 (53.6)
Freq heavy drinking^c^	8.5 (33.3)	16.9 (59.3)	6.7 (33.5)	24.6 (79.0)
Nicotine use	15.5%	27.6%	12.9%	21.0%
Cannabis use^c^	41.9 (108.4)	61.8 (129.4)	12.4 (57.0)	26.2 (86.9)
Illegal drug use	5.5%	3.7%	1.7%	1.7%

*Note:* Numbers represent mean (SD) for continuous variables and percentage of the sample by sex and age group for categorical variables. *Abbreviations*: EUR-like, individuals most genetically similar to reference panels from Europe; AFR-like, individuals most genetically similar to reference panels from Africa; drunk, drunkenness.

a Percentages are based on the analytic sample used in the genetic analyses.

b Relationship quality scores ranged from 0 to 40, with higher scores representing higher relationship quality.

c Alcohol and cannabis use for both participants and partners measured in days per year.

d The emotional well-being variables were assessed using the NIHTB-EB, and were completed by a subset of participants.
