# Supplementary material for: Perceived partner substance use, genetic predispositions, and their associations with problematic alcohol use, emotional well-being, and relationship quality
Source: Psychol Med. Author manuscript; Available in PMC 2026 May 15. (PMC13040299; doi:10.1017/S0033291726103237)

Supplemental Figures

Figure S1. Conditional plot of the interaction between frequency of partner heavy drinking and PGS_AlcCon_ on participant’s frequency of heavy drinking by parenthood.


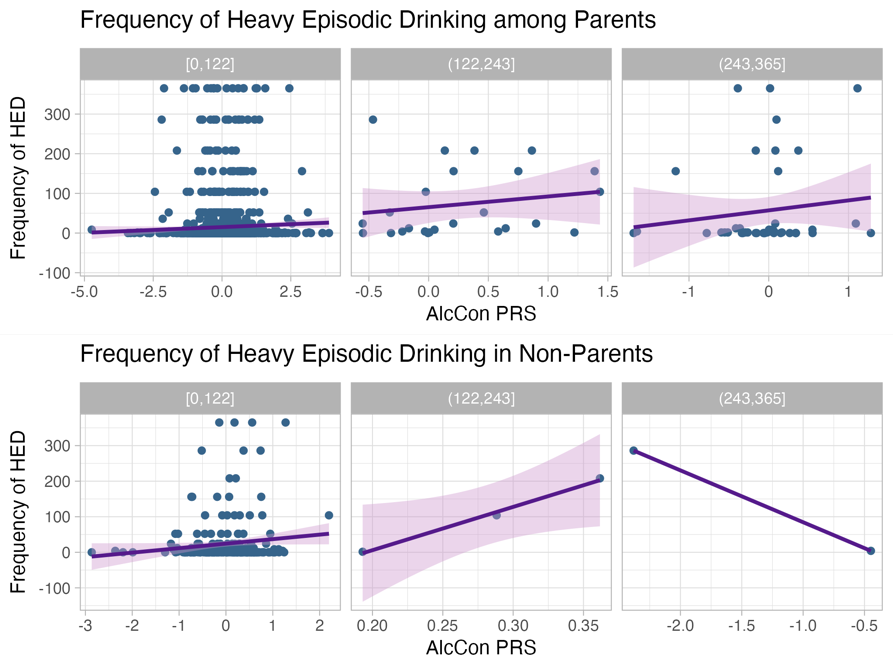


Figure S2. Conditional plot of the interaction between partner past year nicotine use and PGS_AlcCon_ on participant’s frequency of heavy drinking by sex.


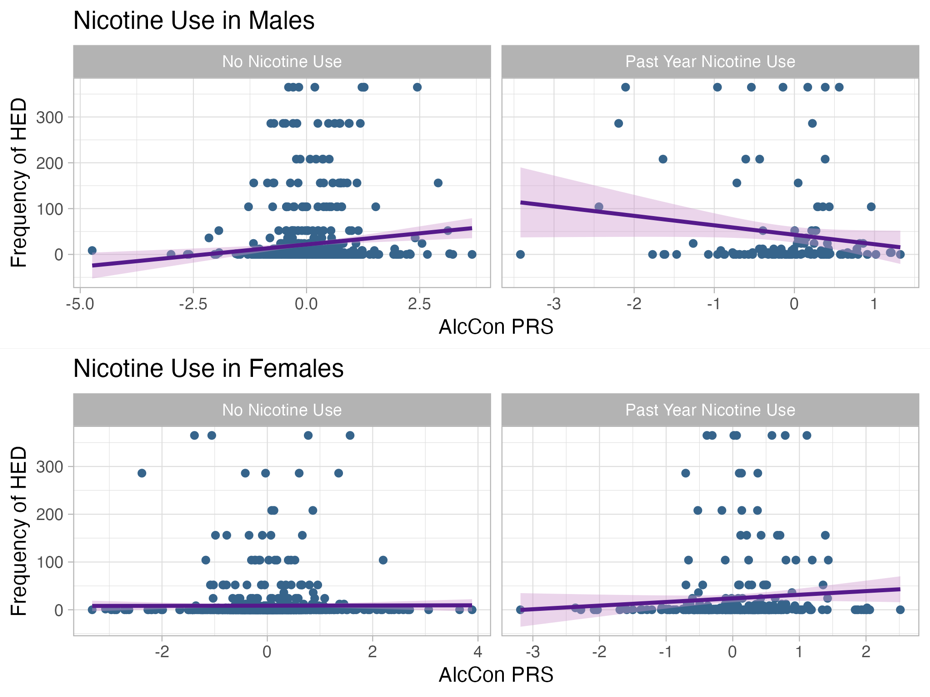

Supplement: Supplement 1 [file NIHMS2168115-supplement-Supplement_1.docx]
